# The Genus *Spilanthes* Ethnopharmacology, Phytochemistry, and Pharmacological Properties: A Review

**DOI:** 10.1155/2013/510298

**Published:** 2013-12-26

**Authors:** Jayaraj Paulraj, Raghavan Govindarajan, Pushpangadan Palpu

**Affiliations:** ^1^Department of Pharmacy, Periyar Maniammai University, Vallam, Thanjavur, TamilNadu 613403, India; ^2^Pharmacognosy and Ethnopharmacology Division, National Botanical Research Institute, Lucknow 226001, India; ^3^Amity Institute of Herbal Product Research, Trivandrum, Kerala 695005, India

## Abstract

*Spilanthes* spp. are popular, over-the-counter remedies; they are sold over the internet under various names and are widely used in traditional medicine in various cultures. This review will summarize the important reports on the ethnopharmacology, botany, phytochemistry, and pharmacological properties as described in the literature from recent years (1920 to 2013). *Spilanthes* spp. are used for more than 60 types of disorders. They are reported to contain a number of biologically active phytochemicals, although a large number of ethnopharmacological uses have been documented; only a few of these species have been investigated for their chemical and biological activities. The studies are carried out mainly on *Spilanthes* extracts and a few metabolites substantiate the uses of these plants in traditional medicine. Well-conducted pharmacological studies are still needed for several traditional indications, and the mechanisms of action by which the plant extracts and the active compounds exert their pharmacological effects remain to be studied. They are predominantly used as extracts in personal care products, traditional medicines, and the pharmaceutical and culinary areas. Suggestions are made regarding some of the possible mechanisms of action as to how the known compounds may exert their biological activity.

## 1. Introduction

Several species in the genus *Spilanthes* Jacq. are tropical plants and are used extensively in traditional medicine and in flavoring foodstuffs. Most people find the spilanthol-induced tingling of the tongue unpleasant, but when cooked, the plants lose their strong flavor and may be used as a green leafy vegetable. For culinary purposes, a small amount of shredded fresh leaves adds unique flavors to salads. In addition, both fresh and cooked leaves are used in dishes such as stews and soups. There have been significant advances in all aspects of *Spilanthes* research, and an increasing number of commercial *Spilanthes* products have appeared in the market place as personal care products, health care products, and for culinary use. Commercial *Spilanthes* plantations have been established to address the need for sustainable supplies of standardized, high quality raw material. The extensive use of this genus in traditional medicine around the globe has been described in many ethnopharmacological reports. Alongside its traditional applications, the importance of this genus lies in the type of disorders for which preparations of its aerial parts and roots are used. Various plants in the genus are used for anti-inflammatory, hepatoprotective, and diuretic properties and in a wide range of disorders like toothache, diuretic, gastritis, gastric ulcers, mucous membrane inflammation, burns, and wounds [1, 2]. For these purposes, infusions and decoctions are prepared from the aerial parts or roots and administered either orally or topically as compresses or baths. Moreover, many biologically active compounds have been isolated from this genus. In recent decades there is a growing research literature on this genus, mainly for the validation of ethnopharmacological usage.

*Spilanthes* spp. have recently been the object of many claims concerning its medicinal properties. A number of publications have shown that these plant extracts, formulations, and bioactive components have a wide range of potential applications in pharmaceutical and cosmetic industries [3]. The patents on *Spilanthes* products and its formulations are increasing. For instance, in the United States alone, some 30 patents have been registered by the US Patent and Trademark Office since 1976 [4]. *Spilanthes* extracts have found applications in pharmaceuticals as an antitoothache formulation, for pain relief, swelling, and gum infections, periodontosis, and in mouthwashes. For instance, A. Vogel Herbal Remedies in the United Kingdom sells organically grown *S. oleracea* L. plant and leaf extracts in alcohol (67% v/v) as a botanical food supplement [5]. A dermal health compound, oral health tonic, and fungus fighter compound marketed by HerbPharm, USA, contains organically grown *S. acmella* Murray and is recommended for skin care, oral health, and antifungal uses [6]. A. Vogel Australia Pty. Ltd. sells formulations which contain *S. oleracea* extract in the Dentaforce herbal mouth spray which is indicated to assist the treatment of moderate cases of periodontal disease and gingivitis. Dentaforce herbal mouthwash and an aftershave cream are also available. Commercial interest in *Spilanthes* has increased tremendously, as indicated by the number of personal care products in the market in which *S. acmella* flower extract is present. For example, in Gatuline from Gattefosse and Antiwrinkle firming light cream from Laboratories SVR *S. acmella* flower extract is added for for its antiaging properties [7, 8]. Nevertheless, despite the real market opportunities in the medicinal, personal care, and food industries, there has been little scientific research to review the potential uses of this genus. Furthermore, the phytochemical compounds responsible for their alleged properties have not yet been reviewed. As a result, the optimization of scientific technologies for their quality control has been neglected. In addition, there is also a need to identify the lacunas where further research is needed to fill in the knowledge gaps.

## 2. Botanical Aspects

The genus *Spilanthes* Jacq. belongs to the family Asteraceae (formerly Compositae) and has more than 300 species, generally distributed in the tropics [9, 10]. The validity of the different species names of plants in the genus *Spilanthes* was searched in international databases, namely, the International Plant Names Index (IPNI), which generated 343 records, and these were verified. This family is known as the aster, daisy, or sunflower family and is the largest family of flowering plants, in terms of number of species. The name “Asteraceae” is derived from the type genus aster, while “Compositae” an older but still valid name referring to the characteristic inflorescence. These herbs originated in tropical Africa and South America and are widely distributed in the tropics and subtropics, including tropical America, North Australia, Africa, Malaya, Borneo, India, and Sri Lanka [11, 12]. These plants have been popularly called toothache plant because of their traditional use. Other common folkloric names include eyeball plant, spot plant, para cress (after the Brazilian province), Brazil cress, alphabet plant, and Australian cress.

This genus is often confused taxonomically with the genus *Acmella* Rich. ex Pers. and sometimes with the genus *Salmea* DC. Comparative morphological and chromosomal studies suggest that these genera are different. The confusion among these species and the misuse of names are very common among traditional and complementary medicine practitioners, herbal users, and particularly among anonymous information sources on the internet [13]. The similar morphological features and ingredient spectra of these species and even genera were and are the cause for this confusion. The anomalies present various problems, such as a large number of overlapping botanical names, synchronous with the formation of frequent and diverse taxonomic revisions, and reclassifications of individual plants expressed in other taxa. Furthermore the traditional uses of these plants and the properties and effects thereof have produced a lot of confusion among earlier researchers. Subsequent genetic, anatomical, morphological, and phytochemical studies showed that although there are indeed close family ties between *Spilanthes* and *Acmella*, they can be distinguished by at least eight morphological characters and by distinctive chromosomes. The main morphological difference between the genera *Spilanthes* and *Acmella* is that the latter has rayed heads and lacks pappus [14]. Comparative morphological studies showed that *Spilanthes* spp. has discoid heads and *Acmella* spp. have rayed heads. The nature of the head is a good taxonomic character for the identification of species in this taxon. Thus the rayed and nonrayed heads seem to be a very reliable characteristic for the broad classification of the genus *Spilanthes*. In chromosome studies, *Spilanthes* has a chromosome number of 16, whereas *Acmella *has 12 or 13 [15]. Jansen recircumscribed the genus and restored the generic status of *Acmella*, which had long been subsumed to a section under *Spilanthes* by earlier taxonomists. Studies of other genera within the *Heliantheae* suggest that both *Spilanthes* and *Acmella* are allied to *Salmea* in the *Verbesininae*.

The genus *Spilanthes* is represented by six species in India; they are *Spilanthes calva* DC., *S*. *paniculata *DC., *S. radicans* Jacq., *S. ciliata* Kunth, *S. uliginosa* Sw., and *S. oleracea* [16]. Typically, these plants are annual herbs or short lived perennials, approximately a half-meter tall with prostrate or ascending cylindrical hairy stems and simple ovate opposite leaves with stipules. They belong to the family Asteraceae, the tribe *Heliantheae*, and the subtribe *Ecliptinae* and have characteristic of flower heads, which distinguish individual species [17]. The flower heads are either solitary or occur in compact or spreading inflorescences. Aerial parts are usually hairy or woolly, and the plants occur as herbs or shrublets that are sometimes dwarfed and are aromatic; the roots are hairy. The key identification characteristics of the genera *Spilanthes* are head solitary, pappus of stiff awns; achenes monomorphic, rhombic, stramineous, cork like margin at maturity, leaves sessile; heads discoid and corollas white to purplish white [18, 19]. The flowers and leaves have a pungent taste, accompanied with tingling and numbness [20, 21].

## 3. Traditional Medicinal Uses

For this review, the ethnopharmacology and ethnobotanical reports were selected with quantitative data and with special attention to the frequency of citation which prioritization for further possible studies [22]. In the tropics and subtropics, these plants are widely used in traditional medicine. The major use in all these systems of medicine is for toothache where the fresh flower head and/or leaves is chewed or placed in tooth cavities to relieve pain [20, 23–27]. Confusion among various species and the misapplication of names are common place among folk herbalists. Other major traditional uses include the following: in India juice of inflorescence of *S. acmella* is used to treat mouth ulcers, and the dried fruits of *S. calva* are powdered and mixed with coconut oil and then used on boils and wounds [28]. Ethiopian traditional healers use the crushed aerial parts in a paste dressing for external injuries [29]. In Nigeria and Sri Lanka, *S. acmella* and *S. uliginosa* are used as a sialagogue [11, 30]. The aerial parts of *S. africana* DC. are crushed and given orally to induce labour during childbirth in western Uganda [31]. In China, *S. callimorpha* A. H. Moore is used as a fertility regulating agent and for amenorrhea [32]. *S. filicaulis* (Schumach.&Thonn.) C. D. Adams and *S. acmella* are used in the treatment of snake bite and rheumatic fever [33, 34]. Plants of this genus are also used to treat parasitic diseases in different traditional systems of medicine [35–37]. For centuries they have been widely cultivated for horticultural, medicinal, insecticidal, and culinary purposes [15], and the application for this purpose is still widespread in different parts of the world.

The uses of the genus *Spilanthes* in traditional medicine can be generally summarized as follows: (a) oral and throat-related conditions like toothache, tooth decay, tooth infections, sore throats, mouth ulcers, paralysis of the tongue, bleeding from gums, throat complaints, stomatitis, gingivitis, and as a sialagogue; (b) other painful conditions like headache, muscle pain, and rheumatism, rubbed on the skin as a local anesthetic; (c) common cold, fever, and cough; (d) gastrointestinal disorders such as stomach ache, dysentery, gastritis, intestinal diseases, diarrhea, and constipation, as an emetic, for liver trouble, and as a tonic during jaundice; (e) others like diuretic activity and the ability to dissolve urinary calculi as an aphrodisiac, amenorrhea, leucorrhoea, anemia, and fertility regulating agent, on boils and wounds and cuts; (f) as an anti-infective, used as an antibacterial, antifungal, and antiviral, and in tuberculosis and pneumonia; (g) antiparasitic activity like malaria, antitrypanocidal, and other worm infections, for the treatment of head infections accompanied by itchiness and as an insecticidal agent; and (h) as a soup, as a fortifier for infants and to get rid of unpleasant symptoms of an alcoholic hangover. Selected, well-conducted ethnopharmacological surveys are summarized in Table 1.

## 4. Phytochemistry

**Figure 1:**
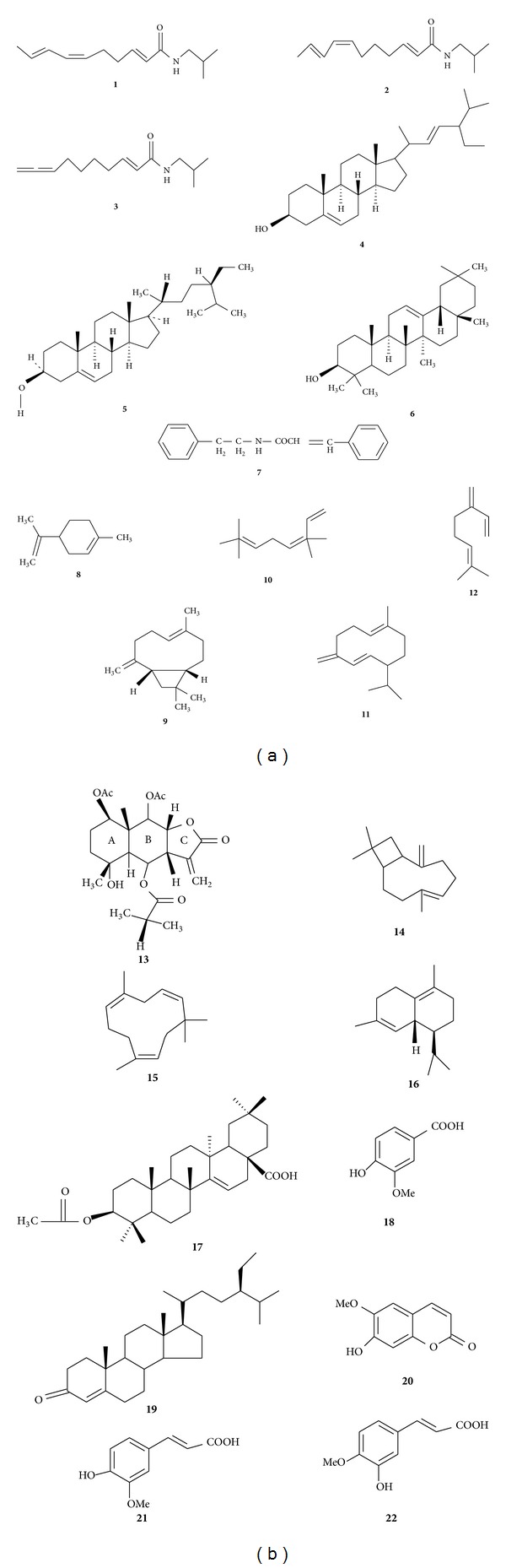


In this genus, the major phytochemicals present are saturated and unsaturated alkyl ketones, alkamides, hydrocarbons, acetylenes, lactones, alkaloids, terpenoids, flavonoids, and coumarins. They are the main constituents considered responsible for the pharmacological activity. Reported chemical constituents from *Spilanthes* are summarized in Table 2 and the compound structures are given in Figure 1. Alkylamides are predominantly found in this genus and have been shown to possess varied biological activities. For instance, they act on cannabinoid type 2 receptor dependent and independent and also have been found to possess useful immunomodulatory effects as chemotaxonomic markers [38]. The principal pungent and bioactive N-isobutylamide compound spilanthol **1** is known to modulate chemosensory receptors and ligands associated with these receptors. Other N-isobutylamides such as undeca-2E,7Z,9E-trienoic acid isobutylamide **2** and undeca-2E-en-8,10-diyonic acid isobutylamide **3** [38, 39]; 2E-N-(2-methylbutyl)-2-undecene-8,10-diynamide; 2E,7Z-N-isobutyl-2,7-tridecadiene-10,12-diynamide; and 7Z-N-isobutyl-7-tridecene-10,12-diynamide from *S. acmella* have been reported [23]. N-Isobutyl-2E,4E,8E,l0Z-dodeca-2,4,8,10-tetraenamide was isolated from aerial parts of *S. mauritiana* [40]. N-Isobutylamides have also been reported in other species, such as *S. alba* [41] and *S. oleracea* [42].

The aromatic amide, N-2-phenylethylcinnamamide **4**, which is rarely found in plants was isolated from the leaves of *S. ocymifolia* [42]. Acetylenes and related compounds were found in *S. alba* L'Herit, *S. americana* Hieron, *S. mauritiana* DC., *S. ocymifolia*, *S. oleracea*, *S. oleracea*, and *S. stolonifera* DC. Myricyl alcohol, pentacyclic triterpenes *α*- and *β*-amyrins, plant sterols *β*-sitosterol **5**, and stigmasterol **6 ** were isolated from the air-dried whole plant of *S. acmella* [43, 44]. From the aerial parts of *S. leiocarpa*, a sesquiterpene *β*-isocomene, lupeol acetate, an epoxide caryophyllene-l, l0-epoxide, alantolactone, onosenlolide, and the sesquiterpene lactone eudesmanolide were isolated [45, 46]. From the leaves of *S. ocymifolia*, stigmasterol, the triterpenoid taraxasterol **7**, and lupeyl acetate were isolated [42]. A new triterpenoidal saponin olean-12-en-3-O-*β*-D-galactopyranosyl (1→4)-O-*α*-L-rhamnopyranoside was isolated from the roots of *S. acmella*. A steiractinolide derivative was isolated from *S. leiocarpa* [47], and eudesmanolide was isolated from the aerial parts of *S. leiocarpa* [46]. Bioassay-guided isolation from *S. acmella* resulted in the isolation of phenolics, vanillic acid **8**, trans-ferulic acid **9**, trans-isoferulic acid **10**, coumarin, scopoletin **11**, triterpenoid 3-acetylaleuritolic acid 12, *β*-sitostenone **13**, stigmasterol, and stigmasteryl-3-O-*β*-D-glucopyranoside, in addition to a mixture of stigmasterol- and *β*-sitosteryl-3-O-*β*-D-glucopyranosides [2]. *S. acmella* flower heads and *S. paniculata* have been reported to contain amino acids [48, 49].

### 4.1. Essential Oil

*Spilanthes* is one of the oil-rich genera belonging to the family Asteraceae, although only a few species have been explored for their essential oils. Most of the investigations studied the essential oils from plants of this genus by conventional methods of analysis, namely, gas chromatography, in most cases coupled with mass spectrometry. The composition of the essential oil is very variable, suggesting the existence of a high number of chemotypes. From the flower heads of *S. acmella* volatile constituents were characterized [50]. In the same plant, the presence of a mixture of C22 to C35 hydrocarbons was also reported [51]. Simultaneous distillation extraction (SDE) and supercritical (CO2) extraction from the flowers, leaves, and stems of *S. americana* resulted in the isolation of volatile metabolites, including sesquiterpenes (*α*- and *β*-bisabolenes, *β*-caryophyllene, *α*-caryophyllene, and an isomeric hydrocarbon cadinene, nitrogenated alkamides (N-(isobutyl)-2E,6Z,8E-decatrienamide; N-(2-methylbutyl)-2E,6Z,8E-decatrienamide; decatrienamide; N-(isobutyl)-6Z,8E-decadienamide; and N-(2-phenylethyl)-2E,6Z,8E-decatrienamide), and oxygenated compounds have been isolated by simultaneous distillation solvent extraction (SDE). Supercritical fluid extraction (SFE) extracts from the stems were found to be rich (>40%) in sesquiterpenes, while those from leaves and flowers were abundant in nitrogenated (43 and 27%) and oxygenated (36 and 23%) compounds [52]. Seven components from the essential oil have been identified, including the sesquiterpene caryophyllene oxide, caryophyllene, limonene, and myrcene as significantly dominating compounds of the essential oil from the inflorescences of *S. calva* DC [53].

## 5. Pharmacological Activities

*Spilanthes* products are sold as over-the-counter herbal medicines and are commonly recommended by traditional healers and used by patients in many countries, derived from raw plant tissue or plant extracts. There are many websites which sell these herbal supplements for their analgesic, antibacterial, and antifungal properties [13]. *S. acmella* is one of the most studied species in this genus from a biological perspective, and biological evaluations are generally based on the traditional uses. Because of the chemical variability of the plants, the main identified compounds are cited when available.

### 5.1. Analgesic and Anti-Inflammatory Activities

Different *Spilanthes* species are used for toothache and sore throat and to relieve pain from boils, cut wounds, and other types of wounds in traditional medicine (Table 1). The analgesic effects were studied using different extracts and animal models. A 100 mg/kg p.o. dose of the ethanol extract of the fresh leaves of *S. acmella* and standard pethidine at 5 mg/kg was used in the tail flick response assay in albino rats to determine central analgesic activity, and significant protection (62.95%) was obtained [54]. The analgesic activity of an aqueous extract of *S. acmella* was tested in the acetic acid-induced writhing response assay in albino mice and the tail flick method in albino rats at doses of 100, 200, and 400 mg/kg. At the end of three hours, the protection from writhing was 46.9%, 51.0%, and 65.6%, respectively [55]. When the isolated compound N-isobutyl-4,5-decadienamide was tested for analgesic activity using the tail flick method in albino rats by applying a thermal method (Hotwire), it showed significant activity in a dose-dependent manner [56]. The aqueous extract of fresh flowers of *S. acmella* showed significant analgesic activity at doses of 111, 335, and 671 mg/kg when administered to male rats. The activity was in a dose-dependent manner, had a rapid onset and a short duration of action, and was not blocked by naloxone, an opioid receptor antagonist. Consequently, it is assumed that analgesic activity is mediated supraspinally accompanied with sedation [57]. Persistent pain attenuation and hyperalgesia by a cold water extract of *S. acmella* flowers were evaluated in rats at doses of 500, 1000, and 1500 mg/kg given orally in the formalin test of nociception and carrageenan-induced thermal hyperalgesia test. The extract showed significant activity in a dose-dependent manner [58].

*Spilanthes* spp. are an important source of anti-inflammatory compounds, and there are numerous studies that validate their use in ethnomedicine for stomatitis, rheumatism, and other painful conditions. A dose of 100 mg/kg p.o. and 500 mg/kg p.o. of an ethanol extract of the fresh leaves of *S. acmella* exhibited anti-inflammatory activity similar to that produced by 100 mg/kg of acetylsalicylic acid (*P* < 0.01). An increase in the tail flick reaction time in albino mice as compared to the control, a peak analgesic effect of the extract, was observed after 90 min of administration [54]. An ethanol extract of the fresh leaves of *S. acmella* was reported to possess potent anti-inflammatory effects in acute (carrageenan-induced), subacute (granuloma pouch), and chronic inflammation (adjutant induced arthritis) models, and in both central (tail flick) and peripheral (glacial acetic acid induced writhing) analgesic assays [54]. An aqueous extract of *S. acmella* was evaluated for anti-inflammatory activity by the carrageenan-induced rat paw edema assay in albino rats, administering the extract at doses of 100, 200, and 400 mg/kg, which showed 52.6%, 54.4%, and 56.1% inhibition of paw edema, respectively. Aspirin at 100 mg/kg p.o. was used as a standard [55]. Extracts of *S. acmella* were obtained by extraction with 85% ethanol, followed by liquid partition against hexane, chloroform, ethyl acetate, and butanol. These fractions were tested for anti-inflammatory activity in the lipopolysaccharide-activated murine macrophage model RAW 264.7. The chloroform extract significantly inhibited nitric oxide production (*P* < 0.01) and was selected for further fractionation to yield the bioactive compound, spilanthol. The diminished levels of LPS-induced inducible nitric oxide synthase (iNOS) and cyclooxygenase (COX-2) mRNA and protein expression support the postulation that spilanthol inhibits proinflammatory mediator production at the transcriptional and translational levels [59].

### 5.2. Antipyretic Activity

In the antipyretic model, an aqueous extract of *S. acmella* at doses of 100, 200, and 400 mg/kg reduced the temperature of pyretic rats significantly from the first hour to the third hour, respectively (*P* < 0.05–0.01). However, the reduction in pyrexia at the fourth hour was not found to be significant, as compared to aspirin [60, 61]. Ethanol and aqueous leaf extracts of *S. acmella* showed significant antipyretic activity in the TAB vaccine-induced pyrexia model, and the extracts exhibited antipyretic activity at a higher dose level 200 mg/kg/p.o. [60].

### 5.3. Antioxidant Activity

The ethyl acetate extract of *S. acmella* showed the most potent antioxidant effect by the DPPH assay. At 200 *μ*g/mL, the ethyl acetate and methanol extracts displayed comparable activity and the highest radical scavenging activity (47.90 and 47.76%) with IC50 values of 216 and 223 *μ*g/mL, respectively 116. The ethyl acetate extract of *S. acmella* exhibits a stronger free radical scavenging capacity than other fractions, as determined by DPPH and ABTS radical scavenging assays. The chloroform extract significantly inhibited nitric oxide production (*P* < 0.01) and for further fractionation yielded the active compound, spilanthol [59]. *In vitro* antioxidant activity of the methanol extracts of the leaves of *S. calva* was studied using various models, including scavenging of ABTS, DPPH, hydroxyl radical, hydrogen peroxide, lipid peroxidation, nitric oxide, and superoxide radical. The methanol extract was found to have significant antioxidant activity [62].

### 5.4. Antiulcer Activity

*Spilanthes* plants are used to treat various types of ulcers, and studies with *S. filicaulis* aqueous extract showed complete mucosal cytoprotection at doses of 500, 1000, and 1500 mg/kg, respectively, in HCl/EtOH-induced gastric lesions in male Wistar rats [63].

### 5.5. Local Anesthetic Activity

The local anesthetic activity of an aqueous extract of *S. acmella* was tested by intracutaneous wheal method in guinea pigs and the plexus method anesthesia in frogs. The test drug, in concentrations of 10% and 20%, produced 70.36% and 87.02% anesthesia, respectively, compared to 97.22% anesthetic effect produced by 2% xylocaine, the standard drug (*P* < 0.001). In the plexus anesthesia model, the time taken by the animals failing to withdraw their feet was recorded as the “onset of local anesthetic action.” A 20% aqueous extract of *S. acmella* and 2% xylocaine were used as standard; the extract showed significant activity (*P* < 0.001). The mean onset of anesthesia with the test drug was faster compared to the standard drug. The onset of local anesthetic activity in the test and standard groups was significantly different from the control group. The anesthetic action of the standard and test drugs continued for 30 minutes. Thus *S. acmella* has a potent local anesthetic effect [61]. Local anesthetic activity is attributed to the presence of the bioactive alkamides in these extracts.

### 5.6. Vasorelaxant Activity

Methanol, chloroform, ethyl acetate, and hexane extracts of *S. acmella* were tested for vasorelaxation on phenylephrine-induced contraction of rat thoracic aorta. Among these, the chloroform extract showed maximum activity; the *R*_max⁡_ of the chloroform extract was 96.6% (ED50 4.28 × 10^−7^) and the ethyl acetate extract exerts immediate vasorelaxation (ED50 76.1 ng/mL). All the tested extracts elicited maximal vasorelaxations in a dose-related manner, although such vasorelaxations are less than those produced by acetylcholine. The vasorelaxation effects of the extracts are completely abolished on the removal of endothelial cells [64].

### 5.7. Diuretic Activity

Based on traditional use, *S. acmella* flower heads were screened for diuretic activity. Oral administration of a cold water extract of 1500 mg/kg in hydrated rats exhibited strong diuretic action and significantly increased in a dose dependent manner. In addition, the extract caused a marked increase in urinary Na+ and K+ levels and a reduction in the osmolarity of urine, suggesting that it is mainly acting as a loop diuretic, which validates the traditional use of this plant [58]. Recent studies on an ethanolic extract of leaves of *S. acmella* at 500 mg/kg body weight p.o. showed significant antidiuretic activity. The extract showed increase in total urine volume and electrolyte excretion of sodium, potassium, and chloride ions. The diuretic activity of the extract may be attributed to its alkaloids, flavonoids, and the presence of mono or divalent salts [65].

### 5.8. Hepatoprotective Activity

A significant hepatoprotective effect has been reported for an ethanolic extract of *S. ciliata* whole plant at different doses, namely, 100, 200, and 400 mg/kg in Wistar rats against paracetamol-induced hepatic damage. This was evident from decreased levels of serum enzymes and an almost normal histological architecture of the liver, following treatment with the plant extract prior to paracetamol treatment. Furthermore, the extract was also effective in increasing the choleretic activity of anesthetized normal rats, and it also shortened hexobarbitone-induced sleeping time in mice, which was increased by carbon tetrachloride treatment, besides showing significant antilipid preoxidant effects *in vitro* [66].

Oral administration of an *S. ciliata* ethanol extract to rats prior to aflatoxin B1 treatment was found to provide significant protection against toxin-induced liver damage, determined [40] hours after the aflatoxin B1 challenge (1.5 mg/kg, i.p.) as evidenced by a significant lowering of the activity of the serum enzymes, and enhanced hepatic reduced glutathione status. Pathological examination of the liver tissues supported the biochemical findings. The plant extracts also showed a significant antilipid peroxidant effect *in vitro* [67].

### 5.9. Antiobesity Properties

Aqueous ethanol (70%) extracts of *S. acmella* flower buds showed pancreatic lipase inhibitory activities in a concentration-(0.75–2.0 mg/mL) dependent manner under *in vitro* conditions. The extract also inhibited lipase, and this plant has potential as a candidate for weight reduction and obesity control [68].

### 5.10. Immunomodulatory Studies

An ethanol extract of the leaves of *S. acmella* was studied for immune stimulatory activity by modulation of macrophage function, carbon clearance assay through Indian ink dispersion in mice (0.5 mL/100 g b.w.i.v.), and for immune prophylactic effects using *E. coli* in mice (0.5 mL/100 g b.w.i.p.). The extract showed significant (*P* < 0.01) peritoneal macrophage stimulation and 25–50% mortality as compared to control mice, indicating its prominent immune stimulant activity [69]. Immunomodulatory activity may be due to the presence of alkamides and polysaccharides in these extracts.

### 5.11. Antimutagenic Studies

A chloroform extract of *S. calva* flower buds produced dose-dependent inhibition of mutagenicity. *S. calva* extract showed more significant inhibition (86.4%) of mutagenesis when evaluated using the Ames salmonella/microsome assay [70]. This activity may be due to the presence of abundant flavonoids and alkamides in these extracts.

### 5.12. Anticancer Activity

*S. spirulina* was tested for anticancer activity (10 *μ*g/mL–5 mg/mL) by tumoricidal effects in an immortal neuroblastoma of spontaneous malignant origin. The findings indicated no pattern of tumoricidal effects with anticancer screen category 5 and hence are considered weak [71].

### 5.13. Metabolic Studies

Ethanol extracts from fresh *S. acmella* were examined with regard to their ability to inhibit cytochrome P4502E1-mediated oxidation of 4-nitrophenol *in vitro*. The alkylamides present in *S. acmella* showed significant inhibition at concentrations as low as 25 *μ*M [72].

### 5.14. Antibacterial and Antifungal Activities

*Spilanthes* spp. are used to treat infections in traditional medicine specifically in the treatment of mouth wounds, boils, cuts, and respiratory tract infections, but only a few species have been established to have good anti-infective properties. Extracts of several species (Table 3) have been subjected to antibacterial testing using a group of randomly selected bacteria, including the Gram-positive bacteria *Bacillus cereus*, *B. pumilus*, *B. subtilis*, *B. cereus*, *Staphylococcus aureus*, *Enterobacter faecalis*, *Pseudomonas aeruginosa*, and *Corynebacterium diphtheriae* and the Gram-negative bacteria *E. coli* by different methods. Among these, the methanol extract of *S. calva* was reported to have significant activity [73]. Agar dilution method assays against strains of microorganisms showed that fractions from the chloroform and methanol extracts weakly inhibited the growth of many tested organisms, for example, *C. diphtheria* NCTC 10356 with minimum inhibitory concentration (MIC) of 64–256 *μ*g/mL and *B. subtilis* ATCC 6633 with MIC of 128–256 *μ*g/mL 2. Aqueous, ethanol, and hexane extracts of *S. americana* were tested against *S. aureus*, *B. cereus*, *Streptococcus hemolyticus*, *E. coli*, *P. aeruginosa*, and the fungus *Candida albicans*. Ethanol extracts were active against all the organisms except *P. aeruginosa*. Hexane extracts were only active against *S. hemolyticus* and *E. coli*, while the aqueous extract was inactive against all the above organisms. None of the three extracts showed antifungal activity [74].

*S. mauritania* roots and flowers were tested against 105 strains of bacteria belonging to seven genera, namely, *Staphylococcus*, *Enterococcus*, *Pseudomonas*, *Escherichia*, *Klebsiella, Salmonella*, and *Mycobacterium* and exhibited an MIC and MBC value >8 mg/mL, but mycobacteria were not inhibited at extract concentrations of 0.5–2 mg/mL [75]. *S. acmella* aqueous alcoholic (90–10%) extracts were found to be inactive against *E. coli* ATCC 25922, *P. aeruginosa* ATCC 15442, *B. subtilis* ATCC 6623, *S. aureus* ATCC 25923, and *C. albicans*, *C. krusei*, *C. parapsilosis*, and *C. tropicalis* [76]. *S. calva* methanol extracts were also inactive [73, 77]. *S. mauritiana* and *S. calva* methanol extracts were inactive against mycobacteria, and it was concluded that the aqueous extracts of this genus are usually microbially inactive [74, 76, 77]. Antimycobacterial activity against *Mycobacterium tuberculosis* H37Ra of the chloroform, methanol, and water extracts of *S. acmella* was studied and found to be less effective, with the MIC being 0.12–1000 *μ*g/mL [78]. A 95% aqueous ethanol extract of *S. acmella* and spilanthol at concentrations of 1 mg, 5 mg, and 10 mg was tested for antibacterial activity against *S. aureus* by the agar diffusion method. All samples were also tested in combination with 5 *μ*g/mL berberine. A 30 *μ*g kanamycin disk was used as a positive control. Spilanthol and the *Spilanthes* extract showed no zone of inhibition and hence no antibacterial activity at that concentration against *S. aureus*. The same samples were also tested with 5 mg/mL berberine and showed no additional inhibition beyond the berberine control [79]. *In vivo* studies showed that spilanthol did not have any antimicrobial activity, whereas the *Spilanthes *extract showed antimicrobial activity, indicating that other metabolites should be examined for this activity. The aqueous, ethanol, and hexane extracts of *S. americana* and the methanol extracts of *S. calva* did not show activity against *C. albicans* and the chloroform, methanol, and water extracts of *S. acmella* whole plants did not show significant activity against *Entamoeba histolytica* at 1,000 *μ*g/mL [73, 74, 77]. Hexane and chloroform extracts of *S. acmella* completely inhibited the growth of *S. cerevisiae* with a MIC 256 *μ*g/mL 2. Petroleum ether extracts of *S. acmella* flower heads were evaluated for antifungal activity against *Fusarium oxysporium*, *Fusarium moniliforme*, *Aspergillus niger*, and *Aspergillus parasiticus* and good inhibition zones were observed against *F. oxysporium* [80]. Extracts from *S. mauritiana* roots and flowers exhibited no activity against *Candida* spp. [81]. *S. iabadicensis* whole plant dichloromethane extracts were reported to have antifungal activity against both *Cladosporium cucumerinum* and *C. albicans* [82].

### 5.15. Antiviral Activity

There are only limited reports regarding the antiviral activity of this genus. Leaves of *S. mauritiana* were explored for antiviral activity with moderate activity shown against HSV, herpes simplex virus, Cox, Coxsackie B2 virus and significant activity against measles, measles edmonston A, polio, poliomyelitis virus type 1 strain 1A/S3,SF, Semliki Forest virus A7 and VSV, and vesicular stomatitis virus T2 [83].

### 5.16. Antiparasitic and Insecticidal Activities

Antiparasitic activity has been widely studied for *Spilanthes* because of its extensive range of use in traditional medicine. This work is summarized in Tables 4 and 5. In sub-Saharan countries these plants are used to treat many parasitic diseases, including malaria and sleeping sickness (Trypanosomiasis), and the plant is also used against various insects. Dichloromethane and methanol extracts of *S. mauritiana* were highly active against *Plasmodium falciparum* strain D [84]. A methanol extract of the flowers of *S. oleracea* showed significant activity against *Trypanosoma brucei* [36, 85]. *S. acmella* extract was investigated for potential larvicide activity [86] and was highly toxic against larvae of *Culex quinquefasciatus* with LC50 value 61.43 ppm [87]. Dichloromethane (1 and 5%) and methanol (1 and 5%) extracts of *S. stolonifera* aerial parts were found to be toxic against *Sitophilus oryzae*. *S. acmella* showed acute toxicity against adult *Periplaneta americana*, and electrophysiological studies indicated immediate hyperexcitation followed by complete inhibition of the cockroach cercal nerve activity [88]. *S. acmella* stimulated cell growth and differentiation of *Herpetomonas samuelpessoai*, a nonpathogenic trypanosomatid, used as biological model because of its similar antigens to *Trypanosoma cruzi*. Crude extracts (1000 *μ*g/mL) or essential oil (250 *μ*g/mL) was added in a defined medium [89]. Cell growth [90] was estimated by counting in Neubauer's chamber, and cell differentiation was examined by light microscopy. *S. acmella* stimulated cell differentiation, which indicates that this plant contains inhibitors against the parasite [76].

## 6. Clinical Trials

A double-blind clinical trial was carried out in Thammasat University Hospital, Thailand, to examine the effects of *S. acmella* in reducing postoperative sore throat soreness after endotracheal intubation. The study used a dose of 180 mg *S. acmella* spray (spilanthol) extract with a sample size of 120 patients. There were no differences in incidence of throat soreness or hoarseness in both recovery (0–2 hr) and postoperative period (24–48 hr), but severity of the sore throat was significantly reduced in the *S. acmella* extract treated group at 0–2 hr after the operation [91].

## 7. Food Preservation

Excellent growth restriction by *S. acmella* extract on the red halophilic cocci isolated from salt cured fish and solar salt was observed. The total viable bacterial count (TVBC) was reported as 4.7, and the total halophile bacterial count was 3.5 (THBC) [139].

## 8. System for Biocontrol of Parasitic Diseases

Extracts of three *Spilanthes* species were used to develop a biocontrol system against the late third and early fourth instar larvae of *A. stephensi *Liston, *A. culicifacies*, *C. quinquefasciatus*, *S. acmella* L. var. *oleracea* Clarke, *S. calva*, and *S. paniculata*; hexane extracts obtained from the flower heads were used. The extracts were potent, with LC50 and LC90 values being 4.57 and 7.83 (*A. stephensi*), 0.87 and 1.92 (*A. culicifacies*), and 3.11 and 8.89 ppm (*C. quinquefasciatus*), respectively. Among the three plant species, *S. acmella* extract proved to be the most effective in inducing complete lethality at minimum dose [140]. *Spilanthol*, at 7.5 ppm concentration, caused 100% motility of eggs, larvae, and pupae of Anopheles, Culex, and Aedes mosquitoes at lower doses; it is also effective against eggs and pupae [141]. The extracts were potent, with LC50 and LC90 values being 4.57 and 7.83 (*A. stephensi*), 0.87 and 1.92 (*A. culicifacies*), and 3.11 and 8.89 ppm (*C. quinquefasciatus*), respectively. Among the three plant species, *S. acmella* extract proved to be the most effective in inducing complete lethality at a minimum dose [140]. Spilanthol found in this genus, at 7.5 ppm concentration, causes 100% motility of eggs, larvae, and pupae of Anopheles, Culex, and Aedes mosquito at lower doses; it is effective against eggs and pupae as well [141].

## 9. Toxicological Considerations

Reports on the toxicity of this genus are very limited. Acute oral toxicity of the essential oil of the flower heads of *S. urens* Jacq. was found to be 2000 mg/kg. Irritability to the eyes, skin, and oral and rectal mucosa was carried out using OECD methods, and it was concluded not to be a potential danger in rats [142]. A quasiexperimental double-blind study conducted in Swiss albino mice studied *S. americana* extract applied topically for a period of 30 days and did not produce any acute toxicity in the tissues of these mice [143]. No adverse effects or mortality were detected in albino rats up to 3 g/kg, p.o. of an aqueous extract of *S. acmella* during the 24 h observation period [61]. The hexane extract of *S. acmella* in male Wistar rats was injected i.p. at 50 to 150 mg/kg b.w. of the extract, and EEG and behaviour were observed for periods up to 2 hours. The lower doses (50 and 75 mg/kg) only elicited minor behavioural changes, such as grooming and wet dog shakes. Higher doses (100 to 150 mg/kg) induced full tonic clonic convulsions in a dose-dependent manner, which were accompanied by typical electrographic seizures in the EEG [144]. The USFDA has added *S. acmella* to the FDA poisonous plant database and listed it as an Indian fish poisons [145]. In Tripura, India, *S. paniculata* leaf paste is added to stagnant water pools for the intoxication of fish in order to capture them easily, and the Miri tribe of Arunachal Pradesh uses coarsely powdered *S. oleracea* whole plant in small ponds and streams and waits for 10–15 minutes before collection of fish [146, 147].

## 10. Biological Activities of Isolated Compounds

Mainly alkylamides are isolated from *S. acmella*, *S. oppositifolia*, *S. oleracae*, *S. mauritiana*, *S. callimorpha*, *S. oleracea*, and *S. ocymifolia* [39, 40, 43, 130, 133–135, 146, 148]. Generally, these alkyl amides are responsible for sialagogue, local anesthetic, analgesic, antibacterial, and antifungal activities. Among these alkyl amides, spilanthol ((E,E,Z)-2,6,8-decatrienoic acid N-isobutylamide) is considered to be one of the most potent alkylamides found in *Spilanthes* spp. Many derivatives of spilanthol have been synthesized and evaluated for their trigeminal sensory properties like burning, pungency, tingling, scratching, numbing, warming, mouthwatering, and cooling effects. Spilanthol was found to be the most active tingling and mouthwatering compound among the natural alkamides present in this genus [148].

Alkyl amides isolated from *Heliopsis longipes* S. F. Blake (Asteraceae) showed good analgesic activity [149], so the ethnopharmacological reports regarding *Spilanthes* analgesic activity may be due to the alkylamides. The mosquitocidal activity was exhibited by dodeca-2E,4E,8E,10Z-tetraenoic acid isobutylamide, an alkamide isolated from *S. mauritiana* [40]. Further studies should be aimed at the validation of traditional uses, including for the treatment of hypertension, peptic ulcer, constipation, infertility, amenorrhea, and alcohol abuse. Other reported compounds are vanillic acid, trans-ferulic acid, scopoletin, 3-acetylaleuritolic acid, *β*-sitostenone, and a mixture of stigmasteryl- and *β*-sitosteryl-3-O-*β*-D-glucopyranosides 2. The above compounds lower the support for the folkloric use. The phytochemistry of this genus needs to be explored in more detail to examine the traditional claims. Pharmacological activities of this genus show inconsistent results because the secondary metabolites vary in extraction method and from time to time. For example, the alkylamides obtained from the different parts of *Echinacea* and *Spilanthes* plants require a variety of extraction methods. Studies have shown that the concentrations of the alkylamides differ among various parts of Echinacea plant; the roots usually contain higher levels than the aerial parts [150]. An HPLC/ES I-MS validated method for the analysis of the alkylamides in *S. acmella* was developed to determine the alkylamide content in the flower heads, leaves, and roots [151].

## 11. Discussion and Outlook

*Spilanthes* spp. extracts and formulations have been used for centuries in traditional medicine in several parts of the world. Different studies, some of them with controversial methodologies, showed that this genus has diverse pharmacological activities and contains several bioactive compounds, although most of them have not been quantified. Several phytochemicals from these plants with supposed pharmacological activities have been identified as possible candidates; these include alkamides, sterols, coumarins, flavonoids, saponins, terpenoids, and polysaccharides. These plants have been reported to contain greater than 0.5% alkylamides, responsible for the local anesthetic, analgesic, antiseptic, sialogogue, and insecticidal properties of this genus which were likely exploited by traditional medicine practitioners [41].

Alkylamides are fatty acid derivatives whose general structure derives from the condensation of an unsaturated or saturated fatty acid and an amine [152]. Alkylamides are known to modulate plant physiology. N-Acylethanolamines (NAEs) are common in both plants and animals. Animal physiology is regulated by low concentrations of long chain polyunsaturated NAEs, and because of their potential role in cell signaling, NAEs and their analogues appear to have diverse activities, with a number of receptor systems, including CB2 and PPAR*γ* [153]. *In vitro* studies of alkylamides have shown various modes of anticancer activity for complex extracts [71]. Another *in vitro* research has shown inhibition of the angiogenesis induced by lung and kidney cancers by an alkylamide containing extract [154]. Structure-activity relationship studies of alkylamides have suggested that they promote differentiation of leukemia to a benign state. Alkylamides at a dose level of 12 mg/kg/day had a significant influence on the phagocytic activity of alveolar macrophages. In addition, the alkylamides caused a dose-dependent increase in NO release from the alveolar macrophages on stimulation with LPS [155]. Recent reports show that these amides demonstrate good bioavailability in humans. Alkylamides also function as cannabinoid receptor 2 (CB2) ligands and have the PPAR receptor and antiviral activity of fatty acid derivatives [156–158]. Alkylamides are abundant in ethanolic extracts that comprise a significant portion of the *Spilanthes* spp. products purchased by patients. These plant products are prepared by maceration or percolation of the starting plant material in a variable ratio of ethanol and water depending on the plant species, plant part, and manufacturing process. Unfortunately, storage conditions are commonly not assessed, which is frequently unreported factor in research investigating medicinal plant efficacy, leaving a gap in the assessment of the research on medicinal plants, and one possible explanation for the inconsistencies in outcomes in such popular herbal remedies such as *Spilanthes* spp. [159]. The traditional healers check the quality of *Spilanthes *spp. extract by the amount of tingling produced by a few drops when placed on their tongue.

Spilanthol, a branched chain, unsaturated aliphatic amide isolated from members of this genus, is a good example of a compound from a traditional medicine which has acted as a drug “lead” in the discovery of many sialagogue compounds, such as N-alkyl-carboxamide derivatives. The presence of flavonoids in this genus [160, 161] justifies its use in traditional medicine for digestive, urinary, analgesic, anti-inflammatory, and skin conditions. The antioxidant effect of these plants may be due to the presence of flavonoids as they act by different mechanisms. For example, some flavonoids inhibit the metabolic activating process of dietary carcinogens, such as aflatoxin Bl. Flavonoids have also been shown to inhibit the cytochrome P450-monooxygenase system, which is involved in the oxidative activation of mutagens. Flavonoids may confer protection through activation of transporters that mediate the extrusion of mutagens from the cell like P glycoprotein and the multidrug resistant protein, MDRP, and detoxifying enzymes like ADPH, quinone reductase, glutathione-S transferase, and so forth. They also block reactive oxygen, nitrogen, and so forth. Metabolites that are involved in mutagenesis, particularly in the context of chronic inflammation, such as flavonoids, inhibit inducible nitric oxide synthase, the main source of NO, and subsequent nitrogen reactive substances in inflammation, and are capable of scavenging these reactive molecules [162–164]. Furthermore, flavonoids have been reported to exhibit a wide range of biological effects, including antibacterial, antiviral, anti-inflammatory, spasmolytic, vasodilator, and hepatoprotective activities. Studies have corroborated that different flavonoid-containing extracts are able to exert an antidiarrheal effect. Thus the antidiarrheal activity of *S. filicaulis* leaf decoction used in traditional medicine and the antibacterial and anti-adhesive properties of these plants may be due to the presence of flavonoids [64, 118, 165–168]. The above pharmacological activities exerted by flavonoids are established *in vitro*, but bioavailability studies did not demonstrate that these compounds reach the particular site to initiate a pharmacological effect, and there is a lack of evidence regarding their bioavailability, *in vivo* enzyme inhibition, pharmacokinetics, and metabolism. Though flavonoids are an important class of compounds, isolation of flavonoids has not been carried out for all *Spilanthes* species, which may be an important area of research, as this will help in understanding the possible mechanism of action of many therapeutic benefits bestowed by these species.

The antipyretic activity of *S. acmella* may be because of the flavonoids present in these plants. Phytochemical studies showed the presence of flavonoids in *S. acmella*. Some flavonoids are predominant inhibitors of either cyclooxygenase or lipoxygenase, so the antipyretic activity may be due to the presence of flavonoids, and again this is proven only *in vitro* and not *in vivo*. Several metabolites in these plants are responsible for the diuretic action, although the contribution level of each one to the total diuretic activity is not yet clear. The main active metabolites that can intervene in diuretic activity are flavonoids, saponins, and monovalent and bivalent cations, but some of these substances could be more active on the glomerular level than on the tubule, provoking an increase in renal circulation and, in this way, glomerular filtration rate and primary urine formation. However, the salts could induce a diuretic effect as a result of an osmotic process. These plants may also act as aquaretics, agents that increase water excretion without affecting renal handling of electrolytes and are unlikely to affect edema or hypertension [169, 170]. Thus, these plants may have potential for the treatment of excessive weight, hypertension, congestive heart failure, kidney stones, and premenstrual syndrome.

The five constituent groups currently believed to be the source of activity in the genus *Spilanthes* spp. are alkamides, coumarins, flavonoids, terpenoids, and polysaccharides. Synergy is often cited as a potential basis of action for many medicinal plant species [171]. Generally, *Spilanthes* spp. extracts are used more than isolated compounds for commercial use for traditional, cosmetic, pharmaceutical, or medicinal purposes. This raises the question of why *Spilanthes* spp., as an extract, is superior to isolated compounds. Considering the number of compounds found in a medicinal plant extract, it seems that either pharmacokinetic potentiation or pharmacodynamic enhancement is highly likely. The presence of plant secondary metabolites in an extract may not be sufficient to exert a pharmacological effect; it depends on the bioavailability of the molecules which reach the target sites to produce a required pharmacological effect. Potential synergies also play a major role in enhancing this effect. The complexity of studying these extracts requires models that can assess multiple pathways simultaneously, since ingestion of a medicinal plant product likely activates multiple pathways. The polyphytochemical nature of these extracts adds to the potential of complex signal transduction, which is difficult to assess in current *in vitro* and *in vivo* models. However, recent technological breakthroughs such as high throughput screening, gene microarrays, and the “omics” platforms in combination with new constructs, such as network pharmacology, offer more realistic biological maps to study these polyphytochemical extracts and to deduce the activity [172]. As traditional medicine continues to increase in popularity, it has become vital to educate the medical and scientific establishments that there are some features which are unique to phytotherapy, and which contribute to both efficacy and safety.

One of these is the concept of synergy, in that a plant extract is more than the sum of its parts, which will substantiate the perception that natural medicines have something special to offer, at least a scientifically based explanation for the clinical bioequivalence of many plant extracts with synthetic drugs for the same therapeutic indications [173]. Market interest in *Spilanthes* spp. products in the area of pharmaceutical, cosmetic, and food industries has a good future, while more studies are needed to identify the potential applications and properties which may explain their mechanisms of action. In order to determine the real potential of these products and to develop new technologies, greater understanding is needed to produce enhanced patient compliance.

## 12. Conclusions

In conclusion, the genus *Spilanthes* offers a wide range of research possibilities. From a botanical point of view, the large numbers of species are confused with other genera and species and require accurate studies to clarify the controversial aspects in the botanical classification of this genus. Without the correct understanding of the genus and species, all of the phytochemical and pharmacological studies will be controversial. The unique morphological diversity of the genus results in a challenging taxonomy, hence accurate botanical identification is important to achieve authentic biological and phytochemical outcomes. Regarding the pharmacological activity of this genus, the studies that have been performed have justified, to some extent, the traditional uses for these plants and also helped to uncover new pharmacological actions. More pharmacological validations are required to support some of the traditional claims. For example, the common name of this species is toothache plant. Therefore, clinical trials to determine its efficacy in tooth decay and tooth infections are essential. Also, pharmacological studies directed at oral infections are desired to validate this popular claim. Microbiological validation against specific mouth organisms *Streptococcus mutans* or *P. gingivalis* is required. The phytochemistry of this genus is complex. So far, the research has been targeted in isolating the alkamides, while more studies are needed to isolate other biologically active compounds. The phytochemistry and biological activity of *S. acmella* have been studied extensively, whilst other species are less studied, and there are no reports on many of the plants in this genus, which is a lacuna in the research on this genus. It is quite often that the flowers are investigated for their biological, chemical, and traditional uses, while the other parts are unexplored. In this genus, biological activity determinations have only targeted alkamides, mainly spilanthol and related amides. Other metabolites should be studied to validate the wide range of traditional uses of this genus. The genus has been found to be rich in coumarins, flavonoids, terpenoids, and polysaccharides, apart from the alkamides. All these groups have significant biological activity which should be studied, validated, and established. Long-term toxicity studies of alkamides are also needed to weigh the activity and toxicity benefits. The studies carried out with the extracts and purified compounds from these plants support many of their reported uses in traditional medicine as an antimicrobial, antifungal, antiviral, and analgesic for the treatment of genitourinary disorders and as an antinociceptive agent. However, well-controlled, double-blind clinical trials are lacking. In many countries, *Spilanthes* spp. products are sold as over-the-counter medicine. Thus a safety evaluation needs to be done on these plants immediately. Well-conducted pharmacological and chemical studies are still necessary for several indications of this species. Its use as an analgesic deserves clinical investigation. It is also astonishing to note that a plant so widely used and also available as over-the-counter medicine has very few or only an isolated clinical trial report. Well-directed clinical trials are required to substantiate the traditional use in order to increase the acceptance and patient compliance.

## Figures and Tables

**Table 1:** Reported uses of genus *Spilanthes* in ethnopharmacological surveys.

Name of the plant	Type of use	Population or geographic zone	Part used and method	References
*Spilanthes acmella * L.	Toothache and throat complaints	India	Flowers and leaves	[23]
*Spilanthes acmella *Murr.	Toothache, insecticidal, colic, gastrointestinal disorders. Dried leaves are strewn around the home to ward off insect pests; a combination of leaf and flower juice is taken for colic	Bangladesh	Flowers and leaves	[92]
	Pain which includes headache, toothache, and muscle pain	Bangladesh		[93]
	Cough	Haryana, India	Whole plant	[94]
	Head infections accompanied by itchiness	Jamalpur District, Bangladesh		[92]
	Toothache	Hasanur Hills, Erode, Tamil Nadu, India	Flowers	[95]
*Spilanthes acmella* (L.) Murray	Anticancer agent	Indonesia	Entire plant Indonesia	[96]

* Spilanthes clava *L.	Toothache and throat complaints	Apatani tribe of Arunachal Pradesh, India	Leaf	[24]
	Teeth were brushed with flowers as remedy for toothache	Bangladesh	Flowers	[92]

* Spilanthes calva* DC.	Toothache and throat complaints	Theni District, Tamil Nadu, India	Flower	[25]
	Dry cough	Tribals of Nandurbar District, Maharashtra, India	Two to three inflorescences crushed and mixed in a spoon with honey taken twice a day for 2-3 days	[97]
	Tuberculosis	Chakma tribe in Hill Tracts Districts of Bangladesh	Root juice	[98]
	Pain	Palani Hills of Tamil Nadu, India	Crushed head inflorescence	[90]

*Spilanthes uliginosa *	Sore throats and gums and in paralysis of the tongue	India	Flower	[20]

* Spilanthes acmella *Murr.	Placed in tooth cavities to relieve pain	Kelantan, Malaysia	Pounded flowers	[26]
	Decotion of roots and leaves is used as gargle for tooth pain	Philippines	Roots and leaves	[99]
	Toothache and dysentery	Saurashtra region, Gujarat, India	Flower	[100]
			
	Leucorrhoea, toothache, anti-inflammatory, astringent, stop bleeding from gums, dysentery, antibacterial, and anemia	Vhabaniganj village, Bogra District, Bangladesh	Leaves and flowers	[101]

*Spilanthes acmella* L.	Ulcer in mouth	Karnataka, India	Juice of inflorescence	[102]

*Spilanthes calva *DC.	Toothache and on boils and wounds	Nagarcoil, India	Fruit dried, powdered, and mixed with coconut oil	[28]
	Dental caries	Nandurbar District, Maharashtr, India	Root and flower head	[103]

*Spilanthes oleracea *	Curing stammering, toothache, stomatitis, and throat complaints	India	Leaves and flowers decoction	[104]

*Spilanthes acmella *	Sialagogue	Sri Lanka	Flowers tincture	[11]

*Spilanthes uliginosa *	Sialagogue	Nigeria and Cameroon	Flower	[30]

*Spilanthes acmella *	Diuretic activity and the ability to dissolve urinary calculi	Uva Province, Sri Lanka	Cold infusion flowers	[11]

*Spilanthes leiocarpa *DC.	Diuretic activity	Andean people of Canta, Lima, Peru	Leaves and flowers infusion	[105]

*Spilanthes calva *	Cuts and mud infection	Bhaudaha, Morang, Nepal	Leaves	[106]
	Cough, cold, and gingivitis	Nawalparasi District, Nepal	Flowers	[107]

*Spilanthes uliginosa *	External injury, crushed paste dressing	Ethiopia	Aerial parts	[29]

*Spilanthes callimorpha *A. H. Moore	Dermatitis	Yunnan Province, China	Aerial part	[108]

*Spilanthes africana *DC.	Induce labor during childbirth	Western Uganda	Aerial part	[31]

*Spilanthes callimorpha *A. H. Moore	Amenorrhea	Hong Kong	Whole plant	[32]

*Spilanthes mauritiana *DC.	Aphrodisiac, treatment of convulsions in children with malaria fever	Mwanga District, Tanzania	Fresh plant	[109]

*Spilanthes filicaulis *	Snakebite	Ghana	Aerial parts	[33]
	Emetic	Benin City, Nigeria	Leaves	[110]

*Spilanthes acmella *L.	Snakebite and rheumatic fever	—	Entire plant	[34]

*Spilanthes caulirhiza *(Delile) DC.	Common cold	Masango, Gabon	Leaves are chewed	[111]

*Spilanthes acmella *Murr.	Fortifier for infants	Madagascar	Leaves soup	[112]

*Spilanthes africana *DC.	Hypertension	Bafia region, Cameroon	Paste mixed with other plants	[113]

*Spilanthes mauritiana *DC.	Convulsions in children malaria, pneumonia, and tonsillitis	—	Leaves	[114]
		Rwanda	Whole plant	[115]

*Spilanthes filicaulis *Jacq.	Peptic ulcer and treatment of tooth decay	Cameroon	Aerial parts	[27]
	Genital infections	Baham, Cameroon	Whole plant	[116]

*Spilanthes acmella *(L.) Murray	Used as an anticancer agent	Indonesia	Entire plant	[117]

*Spilanthes filicaulis *	Intestinal diseases and diarrhoea	Mbalmayo, Cameroon	Leaves	[118]

*Spilanthes paniculata *DC.	Constipation	Apatani tribe of Arunachal Pradesh, India	Leaves	[24]
	Constipation, liver trouble, toothache, worm infection, and as tonic during jaundice	Assam, India	Young stem and leaf	
	For the treatment of intestinal worms, constipation, and toothache	Apatani tribe of Arunachal Pradesh, India	Leaves	[119]

*Spilanthes paniculata *Wall. DC.	Skin disease	Mt.Yinggelin, Hainan Island, China	Flowers	[120]

*Spilanthes paniculata *L.	Toothache, cough, and fever	The Nocte, the Nyishi and the Adi in the Eastern Himalayan region of Arunachal Pradesh, India	Leaf, water decoction	[121]

* Spilanthes paniculata *	Toothache	Tripuri, India	Fresh whole plant	[122]
	Toothache, tooth infections Flowers are chewed followed by closing of the mouth for 5 minutes followed by gargling with water	Barisal District, Bangladesh	Flowers	[123, 124]
	Worm infection	Assam, India	Leaf and flower extracts	[123, 124]
	Decoction of plant is used in dysentery and rheumatism and tincture of flowers relieves toothache	Noakhali District, Bangladesh	Whole plant flowers	[125]
	Cuts	Amazonian Ecuador	Leaves	[126]

*Spilanthes oleracea *Linn.	Malaria	Mali	Flower decoction	[35]

*Spilanthes oleracea* Jacq.	Antitrypanocidal	Mali	Flower decoction	[36]

*Spilanthes filicaulis *	Chest pain, eczema, guinea worm, stomach problems, headache, cough, and toothache; an enema for side pain; used to coagulate blood; rubbed on skin as a local anaesthesia	Cameroon	Entire plant	[37]
	Toothache, stomach ache, gastritis, and malaria	Babungo, Northwest Region, Cameroon	Whole plant	[127]

*Spilanthes callimorpha *A. H. Moore	Fertility regulating agent	China	Aerial part	[32]

*Spilanthes acmella *Murr.	Soup and as a fortifier for infants	Betsimisaraka and Tanala people of Madagascar	Leaves	[112]

*Spilanthes acmella *(L.) Murray	Get rid of unpleasant symptoms of the alcoholic hangover	Brazil	Leaves	[128]

*Spilanthes americana *(Mutis) Hieron	Cough	San Jose Succotz in Belize, Argentina	Prepare a tea from the leaves and drink	[129]

**Table 2:** Reported phytochemicals from genus* Spilanthes. *

Name of the plant	Type of nucleus	Name of the compound	Part used and method	References
*Spilanthes acmella *	Alkamide	Spilanthol **1**, undeca-2E,7Z,9E-trienoic acid isobutylamide **2**, and undeca-2E-en-8,10-diynoic acid isobutylamide **3**, and	Hexane extract of dried flower buds	[39]
*Spilanthes acmella *	Alkamide	Spilanthol **1**, N-2-methylbutyldeca-2E, GZ, 8E-trienamide Q *α* and *β*-amyrm esters and sitosterol-O-*β*-D-glucoslde	Whole plant	[43]
*Spilanthes acmella *	Aliphatic compounds	Lauric, myristic, palmitic linoleic, and linolenic acids as their methyl esters	Whole plant	[130]
*Spilanthes acmella *	Sterols, coumarin	Vanillic acid **18**, * trans*-ferulic acid **21**, scopoletin **20**, 3-acetylaleuritolic acid **17**, *β*-sitostenone **19**, and mixture of stigmasteryl-and *β*-sitosteryl-3-O-*β*-D-glucopyranosides	Aerial parts	[2]
*Spilanthes acmella *	Triterpenoidal saponin	Olean-12-en-3-O-beta-D-galactopyranosyl (1→4)-O-alpha-L rhamnopyranoside	Root	[131]
*Spilanthes acmella *	Long chain 2-ketol ester	Acmellonate N-isobutyl-dodeca-2E,4E,8Z,l 0 E-tetraenamide **3**	Ethyl acetate extract Whole plant	[132]
*Spilanthes oppositifolia *	Alkamide	Spilanthol **1**	Aerial parts	[133]
*Spilanthes alba *	Unsaturated amides	Acetylemc amides	Aerial parts	[134]
*Spilanthes mauritiana *	Alkamide	N-isobutyl-2E,4E,8E.lOZdodeca-2,4,8,1O-tetraenamide	Aerial parts	[40]
*Spilanthes leiocarpa *	Terpeniods	*β* Isocomene, lupeyl acetate, caryophyllen-l,l0-epoxide, and alantolactone, eudesmanolide **13**	Aerial parts	[45]
*Spilanthes ocymifolia *	Amides Terpenoid	N-2-Phenylethylcinnamide, stigmasterol Taraxasterol acetate lupeyl acetate	Leaves	[42]
*Spilanthes callimorpha *	Alkamides	8,11-dihydroxy-dodeca-2E,4E, 9E-triensaureisobutylamide and 7-hydroxy-trideca-2E, 8E-dien-10, 12-diynoic acid isobutylamide	Whole plant	[135]
*Spilanthes oleracea *L.	Alkamides	Z-Non-2-en-6,8-diynoic acid isobutylamide **3** and (Z)-dec-2-en-6,8-diynoic acid isobutylamide	Whole plant	[41]

**Table 3:** Antibacterial and antifungal activities of genus *Spilanthes. *

Classification	Species	Tested material	MIC	Active/inactive	References
Gram +	*Staphylococcus aureus *	*Spilanthes calva* methanol extract	8 *μ*g/mL	Active	[73]
Gram +	*Bacillus subtilis *	*Spilanthes calva* methanol extract	8–9.9 *μ*g/mL	Active	[73]
Gram +	*Bacillus subtilis *ATCC 6633	Fraction of chloroform extract *Spilanthes acmella* Murr.	128 *μ*g/mL	Active	[2]
Gram +	*Bacillus subtilis *ATCC 6633	Fraction of chloroform extract *Spilanthes acmella* Murr.	128 *μ*g/mL	Active	[2]
Gram +	*Bacillus subtilis *	*Spilanthes calva* Methanol extract	8.0–9.9 *μ*g/mL	Active	[73]
Gram +	*Bacillus cereus *	Fraction of chloroform extract *Spilanthes acmella* Murr.	256 *μ*g/mL	Active	[2]
Gram +	*Corynebacterium diphtheriae *NCTC 10356	Fraction of chloroform extract *Spilanthes acmella* Murr.	64 *μ*g/mL	Active	[2]
Gram +	*Staphylococcus epidermidis *ATCC 12228	Fraction of chloroform extract *Spilanthes acmella* Murr.	128 *μ*g/mL	Active	[2]
Gram +	*Enterobacter faecalis *	*Spilanthes clava* methanol extract	—	Inactive	[73]
Gram +	*Mycobacter phlei *	*Spilanthes clava* methanol extract	—	Inactive	[73]
Gram +	*Micrococcus luteus *ATCC 10240	Fraction of chloroform extract *Spilanthes acmella* Murr.	128 *μ*g/mL	Active	[2]
Gram +	*Staphylococcus epidermidis *ATCC 12228	Fraction of chloroform extract *Spilanthes acmella* Murr.	128 *μ*g/mL	Active	[2]
Gram +	*Streptococcus pyogenes *II	Fraction of chloroform extract *Spilanthes acmella* Murr.	256 *μ*g/mL	Active	[2]
Gram +	*Micrococcus luteus *ATCC 10240	Fraction of chloroform extract *Spilanthes acmella* Murr.	128 *μ*g/mL	Active	[2]
Gram +	*Streptococcus mutans *	Chloroform extract of *S. acmella *	250 *μ*g/mL disk (7.5 mm zone)	Active	[136]
Gram −	*Escherichia coli *	*Spilanthes calva* methanol extract	—	Inactive	[73]
**Fungi**	*Candida albicans *	*Spilanthes calva* methanol extract	—	Inactive	[73]
	*Saccharomyces cerevisiae *ATCC 2601	Fraction of chloroform extract *Spilanthes acmella* Murr.	256 *μ*g/mL	Active	[2]
	*Saccharomyces cerevisiae *ATCC 2601	Hexane extract *Spilanthes acmella* Murr.	256 *μ*g/mL	Active	[2]

**Table 4:** Antiparasitic activities of extracts of *Spilanthes*.

Classification	Species	Tested material	MIC (*μ*g/mL)	Other results	References
*Trypanosoma *	*Trypanosoma brucei *	Dichloromethane extract Flowers of *S. oleracea* Jacq.	100	Active	[85]
*Trypanosoma *	*Trypanosoma brucei* STlB 345	Flowers of *Spilanthes oleracea* methanol extract	10	Active	[36]
*Plasmodium *	*Plasmodium falciparum *strain D10	Cold DCM extract of stems of *S. mauritiana *(Pers.) DC.	38	Active	[84]
		Dichloromethane and methanol *Spilanthes mauritiana* (Pers.) DC.	5.3	Active	

**Table 5:** Insecticidal activities of extracts of *Spilanthes*.

Classification	Species	Tested material	Results of the test	References
*Arthropoda*	*Sitophilus Oryzae *	Dichloromethane (1 and 5%) and methanol (1 and 5%) extract of *S*. *stolonifera* DC. aerial part	Toxic	[89]
*Arthropoda*	Larvae of Culex *quinquefasciatus *	*S. acmella *	LC50 value 61.43 ppm	[87]
Glyprhelrnins	*Physa occidentalis *	*S. oleracea *	50 (LC100) ppm	[137]
*Arthropoda*	*Periplaneta americana* adult	*S. acmella* Murr.	Acute toxic	[88]
Cypriniformes	*Danio rerio* embryos	*Spilanthes acmella* (Linn.) Murr. 20% aquous extract	No lethel effect	[138]
